# Citrus Flavonoids for Cystic Fibrosis Treatment

**DOI:** 10.1002/cbic.202500586

**Published:** 2025-10-10

**Authors:** Mario Pagliaro, Caterina Di Sano, Claudia D'Anna, Giovanna Li Petri, Giuseppe Angellotti, Rosaria Ciriminna

**Affiliations:** ^1^ Istituto per lo Studio dei Materiali Nanostrutturati CNR via U. La Malfa 153 90146 Palermo Italy; ^2^ Istituto per la Farmacologia Traslazionale CNR via U. La Malfa 153 90146 Palermo Italy

**Keywords:** anti‐inflammatory, antibacterial, CFTR stimulation, citrus flavonoids, cystic fibrosis

## Abstract

Studies conducted since the late 1990s show substantial activity of selected citrus flavonoids as cystic fibrosis transmembrane conductance regulator (CFTR) membrane protein activators. Aimed at reinvigorating fundamental and applied research on citrus flavonoids for the treatment of mucoviscidosis, the thesis of this work is that aptly formulated citrus flavonoids hold significant potential for developing a multitarget treatment of cystic fibrosis combining in a single treatment antimicrobial, anti‐inflammatory, CFTR‐stimulating, and immunomodulatory properties, as required by a genetic disease eventually causing chronic lung inflammation and bacterial infection reinforcing each other.

## Graphical abstract

Studies conducted since the late 1990s show substantial activity of selected citrus flavonoids as cystic fibrosis transmembrane conductance regulator (CFTR) membrane protein activators. The thesis of this work is that aptly formulated citrus flavonoids hold sigificant potential for developing a multitarget treatment of cystic fibrosis combining in a single treatment antimicrobial, anti‐inflammatory, CFTR‐stimulating, and immunomodulatory properties, as required by a genetic disease eventually causing chronic lung inflammation and bacterial infection reinforcing each other.

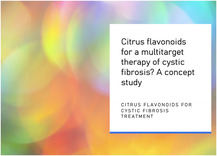

## 1 Introduction

Also called since 1944 mucoviscidosis as the body makes thick and sticky mucus that can cause damage throughout the body, including airways and lungs, cystic fibrosis (CF) is a autosomal recessive condition chiefly affecting Caucasian populations.^[^ ^1^ ^]^ The disease is caused by mutations in the gene providing instructions for synthesizing the cystic fibrosis transmembrane conductance regulator (CFTR) protein chloride transport channel.^[^ ^2^ ^]^

In brief, reduced activity of mutant CFTR gene causes CF. Mucociliary clearance, airway's main defense against airborne disease, depends on proper function of the CFTR chloride transport channel. Activated by a protein kinase (PKA‐C) the CFTR channel mediates salt‐water transport across epithelia acting as a cyclic adenosine monophosphate (cAMP)‐regulated chloride channel at the apical membrane of airway epithelial cells.^[^ ^3^ ^]^ cAMP is a intracellular messenger molecule synthesized from ATP by the enzyme adenylyl cyclase involved in various cellular processes, including relaying signals from outside the cell to the inside.

When the channel is not functional, as it happens in CF patients, dysfunctional CFTR leads to a dehydrated airway surface layer and buildup of sticky mucus eventually leading to growth of pathogenic bacteria and infection.

CF is often lethal due to chronic bronchopulmonary infections (especially chronic *Pseudomonas aeruginosa* infection) with pulmonary insufficiency as the main cause of death.^[^ ^2^ ^]^ In CF patients infection and inflammation establish a vicious circle of infection and inflammation, with each exacerbating the other, causing progressive and irreversible lung damage.^[^ ^4^ ^]^

Most pharmacological approaches targeting CF were directed at correcting the defect in ion transport by stimulating chloride secretion.^[^ ^5^ ^]^ Thanks to better symptomatic treatments with anti‐inflammatory and antibiotic drugs, as well as to the introducion of new CFTR modulator drugs in the 2010s, life expectancy has improved, especially in high income countries.^[^ ^6^ ^]^ Yet, as lately noted by Addissouky and coworkers, symptomatic treatments remain essential, because pre‐existing organ damage cannot be reversed by CFTR modulators.^[^ ^7^ ^]^

The use of natural products as antifibrotic drugs is particularly promising. Fibrosis indeed develops from chronic inflammation, myofibroblast activation to epithelial‐mesenchymal transition (EMT), and extracellular matrix accumulation (ECM). Both EMT and ECM can be inhibited by certain natural products, thereby inhibiting fibrosis in one organ, simultaneously targeting fibrosis in multiple other organs.^[^ ^8^ ^]^

Virtually insoluble in water and chiefly consisting of flavanones, flavones, and flavonols (and anthocyanins, in red oranges), citrus flavonoids are amid said natural products. Polyphenolic molecules having a phenylpropanoid chain (C6–C3–C6) basic structure consisting of two aromatic rings (A and B) linked by a heterocyclic pyran ring (C), the most common flavonoids in citrus fruits include quercetin, naringin, naringenin, hesperidin, neohesperidin, hesperetin, nobiletin, rutin, luteolin, diosmin, apigenin, and kaempferol. Concentrated in the fruit peel and to a lower extent present also in the pulp, these flavonoids exert significant health beneficial properties,^[^ ^9^ ^]^ including anti‐inflammatory activity.

Showing first evidence of their potential in management of CF, Illek and Fisher reported in 1998 of citrus flavonoids such as apigenin, kaempferol, and quercetin to activate CFTR‐mediated Cl^−^ currents both in vivo and in vitro*.* ^[^ ^10^ ^]^ The same team in 2000 reported that apigenin toos is an activator of cystic fibrosis (CFTR)‐mediated Cl^−^ currents across epithelia at low concentrations and a blocker at high concentrations, that likely binds to a stimulatory and an inhibitory binding site, differentiated by their molecular interactions during binding.^[^ ^11^ ^]^

Regardless of subsequent promising results reported in the subsequent 25 years using different citrus flavonoids (see below), in the last decade (2015–2024), only a few studies focused on the use of citrus flavonoids for treating CF. On the other hand, in the same time period, a signficant number of studies unveiled the potential of flavonoids, many of which citrus flavonoids, in the treatment of pulmonary fibrosis (PF).^[^ ^12^ ^]^ PF is another chronic fibrotic pathology whose causes, though, are not genetic.

Aimed at progressing and stimulating new fundamental and applied research on citrus flavonoids for the treatment of cystic fibrosis, this account identifies the main findings emerged in the pioneering studies. Aptly formulated, we conclude, citrus flavonoids hold potential to develop a multitarget treatment of CF combining in a single treatment antimicrobial, anti‐inflammatory, CFTR‐stimulating, and immunomodulatory properties, as required by a genetic disease eventually causing chronic lung inflammation and bacterial infection reinforcing each other.

## 2 Citrus Flavonoids and Cystic Fibrosis

### 2.1 Methodology

A Boolean search was conducted by mid 2025 on research database Google Scholar with the queries ‘citrus flavonoids’ + ‘cystic fibrosis’. The search returned 83 research or review articles in English.^[^ ^13^ ^]^ The indexed articles identified were inspected individually excluding those not relevant to the topic of the present study. **Table** **1** lists the main articles identified alongside the year of publication and the country where research was conducted.

**Table 1:** Research articles on citrus flavonoids for cystic fibrosis treatment [source: Google Scholar, 2025].

Article title	Year of publication	Country	Reference
Flavonoids stimulate Cl conductance of human airway epithelium in vitro and in vivo	1998	USA	[10]
Structural determinants for activation and block of CFTR‐mediated chloride currents by apigenin	2000	USA	[11]
Activation of the Cystic Fibrosis Transmembrane Conductance Regulator by the Flavonoid Quercetin	2010	USA	[18]
Hesperidin stimulates cystic fibrosis transmembrane conductance regulator–mediated chloride secretion and ciliary beat frequency in sinonasal epithelium	2010	USA	[21]
Quercetin increases cystic fibrosis transmembrane conductance regulator‐mediated chloride transport and ciliary beat frequency: therapeutic implications for chronic rhinosinusitis	2011	USA	[19]
Leucine enhances aerosol performance of naringin dry powder and its activity on cystic fibrosis airway epithelial cells.	2011	Italy	[22]
The activation effect of nobiletin on cystic fibrosis transmembrane conductance regulator chloride channel	2013	China	[15]
Stimulation effect of wide type CFTR chloride channel by the naturally occurring flavonoid tangeretin	2014	China	[16]
Nobiletin stimulates chloride secretion in human bronchial epithelia via a cAMP/PKA‐dependent pathway	2015	Hong Kong	[17]
Naringenin regulates CFTR activation and expression in airway epithelial cells	2017	China	[23]

Kaempferol, quercetin, hesperidin, naringenin, naringin, apigenin, nobiletin, and tangeretin are the first citrus flavonoids for which promising activity has been reported so far, in in vitro and in vivo studies published between 1998 and 2017. In the following, we summarize the main findings identified in the pioneering studies, presenting results according to the flavonoid class. We briefly remind that flavonoids are heterocyclic compounds whose classification into flavonol, flavone, flavoanone, anthocyanin, and isoflavanone subgroups is based on cyclization and degree of oxidation in the C3 chain.^[^ ^14^ ^]^ Flavones and flavonols have a double bond at C2 and C3, while flavonols are hydroxylated at C3 position. After oxidation, they respectively become their respective dihydroxylated forms: flavanones and flavanonols. Polymethoxyflavones (PMFs) are further distinguished from other flavonoids by their structure, which includes more than one methoxy group attached to the flavonoid skeleton.

### 2.2 Flavones: Apigenin, Nobiletin, and Tangeretin

In the first study showing the capacity of citrus flavonoids, and of flavones in particular such as apigenin (4′, 5,7‐trihydroxyflavone) dissolved in dimethylsulfoxide (DMSO), to activate CFTR‐mediated Cl^−^ currents, both in vivo and in vitro, in 1998, Illek and Fisher used single Calu‐3 cells (a human airway cell line of adenocarcinoma origin), transepithelial measurements in Calu‐3 monolayers, and in vivo measurements of nasal potential difference.^[^ ^10^ ^]^

The team showed that apigenin 30 μM was the most effective flavonoid in stimulating maximal current (normalized to forskolin‐stimulated currents) in a rank series of apigenin ≥ kaempferol ≥ genistein = quercetin in both experiments with whole cells and in transepithelial experiments. The latter recordings were significantly smaller than those in whole cell recordings, suggesting that, in an intact epithelial setting, currents were limited by other factors.

**Figure 1:**
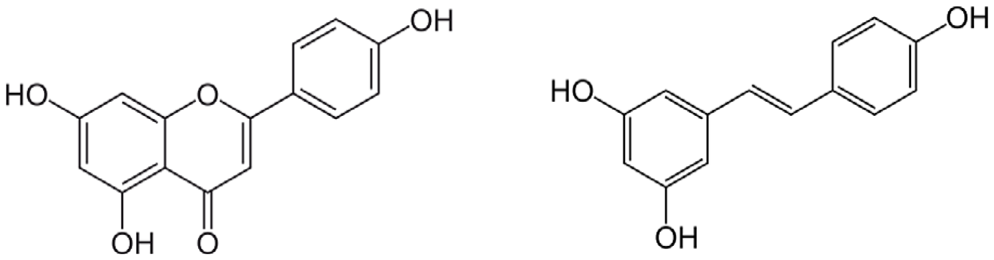
Structures of apigenin (*left*) and *trans*‐resveratrol (*right*). Numbers of substituents refer to the numbering of the basic flavone structure.

Two years later, the same team reported that apigenin too is a CFTR activator at low concentrations and a blocker at high concentrations.^[^ ^11^ ^]^ In an elegant pharmacological study, the roles of the 3 hydroxyls of apigenin in stimulating Cl^−^ currents were investigated by removing single or all hydroxyls or by methoxylating single or all 3 hydroxyl positions. Apigenin‐related stilbene *trans*‐resveratrol was included to test the effect of the central pyrone ring structure of apigenin (**Figure** **1**).

**Figure 2:**
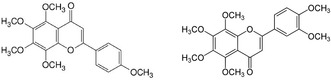
Structures of tangeretin (*left*) and nobiletin (*right*).

Between 2013 and 2014, scholars in China led by Yang reported that both nobiletin^[^ ^15^ ^]^ and tangeretin,^[^ ^16^ ^]^ two flavones further characterized by being also polymethoxyflavones (PMFs, **Figure** **2**), are CFTR activator whose activation effect on CFTR chloride channel involves direct interacton with the protein. Tangeretin (4^′^, 5,6,7,8‐pentamethoxyflavone) is a PMF particularly abundant in the peel of *Citrus aurantium*, whereas nobiletin (5,6,7,8,3′, 4′‐hexamethoxyflavone) is abundant in the tangerine (*Citrus reticulata*) peel.

In both cases, the team concluded that two flavonoids might have potential use for the treatment of CFTR‐related diseases like cystic fibrosis and bronchiectasis.

In 2015, researchers led by Ko in Hong Kong reported that nobiletin stimulates chloride secretion in human bronchial epithelia via a cAMP/PKA‐dependent pathway.^[^ ^17^ ^]^ In further detail, the activation of apical CFTR chloride channels mediated by nobiletin involves stimulating adenylate cyclase and the cAMP/PKA pathway through direct binding.

Nobiletin dissolved in cell culture medium at 100 μM load stimulated a real‐time increase in intracellular cAMP but not Ca^2+^ in 16HBE14o‐ human bronchial epithelial cell line. To further confirm that the nobiletin‐stimulated Cl^−^ secretion was due to the activation of CFTR, researchers used the CF cell line (CFBE41o‐) lacking functional CFTR. No increase in short circuit current (*I*_SC_) could be observed after the application of apical nobiletin (100 μM). For CFBE41o‐ cells grown on glass coverslips, the addition of 100 μM nobiletin also increased intracellular cAMP levels, similar to that observed in normal 16HBE14o‐ cells. However, the application of calcium‐mobilizing agent UTP stimulated an increase in *I*_SC_, which was due to the activation of CaCC.^[^ ^17^ ^]^

These results were consistent with previous findings reported in a journal published in Chinese by Yang and coworkers in 2013 wherein the team had reported that nobiletin activates CFTR chloride channel through a direct binding way.^[^ ^15^ ^]^ The same team the subsequent year reported that tangeretin too is an activator of the CFTR chloride channel activation whose mechanism involves direct interacting with the protein.^[^ ^16^ ^]^ In detail, the effect of tangeretin on CFTR channel activity was confirmed by excised inside‐out patch clamp studies. ATP (1 mM) and PKA (25 UM) were added into the system to activate the channels. After the activation, patches were exposed to 1 mM ATP plus indicated concentrations of tangeretin. Tangeretin (20  and 40 μM) increased CFTR Cl^−^ currents by 70 and 120%, respectively. The activation was reversible, as currents recovered after removal of tangeretin.

### 2.3 Flavonols: Quercetin and Kaempferol

**Figure 3:**
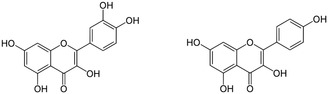
Structures of quercetin (*left*) and kaempferol (*right*).

As mentioned above, in the first study showing the capacity of citrus flavonoids to activate CFTR‐mediated Cl^−^ currents, both in vivo and in vitro, Illek and Fisher in 1998 reported that, besides flavone apigenin, also kaempferol and quercetin (two flavonols, **Figure** **3**) stimulated Cl^−^ currents in Calu‐3 cells and in vivo measurements of nasal potential difference dose dependently.^[^ ^10^ ^]^

Quercetin (at concentrations > 40 μM, equivalent to 12.1  μg ml^−^^1^) stimulated Cl^−^ secretion in vitro, whereas higher doses were inhibitory. Stimulation of cell monolayers with forskolin significantly increased their sensitivity to flavonoids reducing their half‐maximal stimulatory concentrations: kaempferol (2.5  ±  0.7 μM) ≤ apigenin (3.4  ± 0.9 μM) ≤ quercetin (4.1 ± 0.7 μM) ≤ genistein (6.9 ± 2.2 μM). This outcome indicates that binding of the flavonoids is dependent on the level of stimulation of the cAMP‐PKA system by forskolin. Furthermore, activation kinetics of currents showed cooperative binding of flavonoids at two (or more) distinct sites, leading the team to conclude that ‘the cooperative binding of flavonoids happens at the two nucleotide binding domains in a low‐phosphorylation mode, which, after phosphorylation of CFTR, increase their affinity and lose their cooperativity’.^[^ ^10^ ^]^

In 2010, Rowe and coworkers confirmed that quercetin dissolved in DMSO activates CFTR‐mediated anion transport in respiratory epithelia in  vitro and in  vivo and demonstrated that quercetin indeed does not produce detectable phosphorylation of the isolated CFTR R‐domain.^[^ ^18^ ^]^ In detail, using CFBE41o‐ human CF bronchial epithelial cells (a cell line derived from a cystic fibrosis patient homozygous for the ΔF508 CFTR mutation) results showed that quercetin produced a small increase in cAMP relative to forskolin, despite potent stimulation of ion transport compared with cAMP agonist. However, quercetin had no detectable effect on R‐domain phosphorylation at any concentration tested.

This outcome markedly differs from what originally found by Illek and Fisher with quercetin tested on Calu‐3 cells who demonstrated a cAMP/PKA‐dependent activation of CFTR.^[^ ^10^ ^]^

Rowe and coworkers proposed a CFTR activation mechanism independent of PKA and R‐domain phosphorylation producing a dose‐dependent hyperpolarization of the nasal potential difference also in normal human subjects.^[^ ^18^ ^]^ The team concluded that quercetin may provide two stimuli that together optimize ΔF508‐CFTR activation (including both cAMP‐dependent and CAMP‐independent effects), particularly in cells where endogenous cAMP levels could be limiting.

In the following year (2011), the same team reported that quercetin dissolved in DMSO is an activator of ciliary beat frequency (CBF) in primary cultures derived from individuals with functional CFTR but not in cultures from patients homozygous for the *Δ*F508 mutation.^[^ ^19^ ^]^ In detail, quercetin increased CBF in normal but not in CF human sinonasal epithelial cultures.

Cultures derived from individuals with functional CFTR had a modest, but significant increase in CBF after application of quercetin (10 μM) when compared with DMSO control (1.37 vs. 1.24 respectively). Analogous to explants in CFTR−/‐ mice, quercetin did not augment CBF in homozygous *Δ*F508 HSNE cells, namely the human small airway epithelial cells (HSNE) that carry the *Δ*F508 mutation in the CFTR gene. This outcome led to the team to suggest to use quercetin in topical administration to the sinuses of individuals with relative deficiency in CFTR activity ^[^ ^19^ ^**]**^

### 2.4 Flavanones: Hesperidin, Naringin, and Naringenin

**Figure 4:**
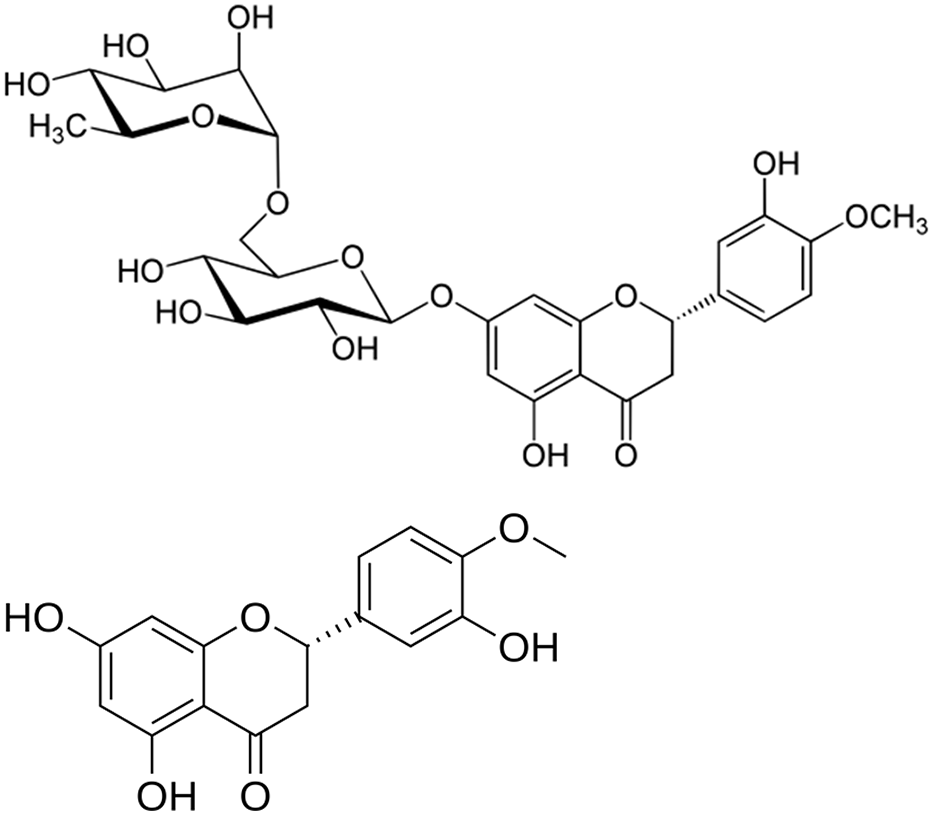
Structures of hesperidin (*top*) and hesperetin (*bottom*).

Imparted with numerous therapeutical effects in various diseases, such as neurological disorders and cardiovascular diseases, due to its anti‐inflammatory, antioxidant, lipid‐lowering, and insulin‐sensitizing properties, hesperidin (hesperitin, 7‐O‐rutinoside) along with its aglycone hesperetin ((2*S*)‐3′, 5,7‐trihydroxy‐4′‐methoxyflavan‐4‐one, **Figure** **4**) is the most widely studied citrus flavonoid in biomedicine.^[^ ^20^ ^]^

Using both murine and human nasal airway cells in vitro, in 2010, Woodwarth and coworkers at the University of Alabama reported that hesperidin dissolved in DMSO stimulates both CFTR–mediated chloride secretion and CBF in sinonasal epithelium.^[^ ^21^ ^]^

In detail, CBF was significantly increased when compared to the control vehicle (phosphate‐buffered saline) when hesperidin 1 mM was applied to the basal media, whereas the PKA inhibitor H‐89 significantly decreased hesperidin‐stimulated CBF under all conditions.

Hesperidin exhibited several unique properties in comparison to quercetin. While quercetin and other flavonoids have characteristic inhibitory effects at higher concentrations,^[^ ^10^ ^]^ hesperidin due to decreased solubility at higher concentrations did not drive inhibitory effects. Furthermore, CFTR activation was weaker in MNSE but more active in HSNE, while in vivo CFTR stimulation was comparable to forskolin (according to murine NPD measurements). In vitro data suggest that hesperidin can stimulate nearly all available CFTR‐mediated Cl^−^ transport in HSNE.^[^ ^21^ ^]^

Likewise to quercetin, hesperidin activity as a CFTR channel potentiator was ascribed to its interactions with the nucleotide binding domains of CFTR protein, rather than phosphorylation of the regulatory domain (R‐D). Hesperidin indeed had no detectable effects in an assay of R‐D phosphorylation of polyclonal NIH‐3T3 cells expressing HA‐tagged R‐domain treated with hesperidin and compared to forskolin and DMSO control. Phosphorylation results in a 2–4 kD shift in migration that took place only using forskolin. Furthermore, hesperidin also had no detectable effect on cellular cAMP concentrations when compared to DMSO control, whereas marked cAMP enhancement was driven by forskolin used as positive control. The team indeed concluded that this finding had therapeutic implications for chronic rhinosinusitis.^[^ ^21^ ^]^

**Figure 5:**
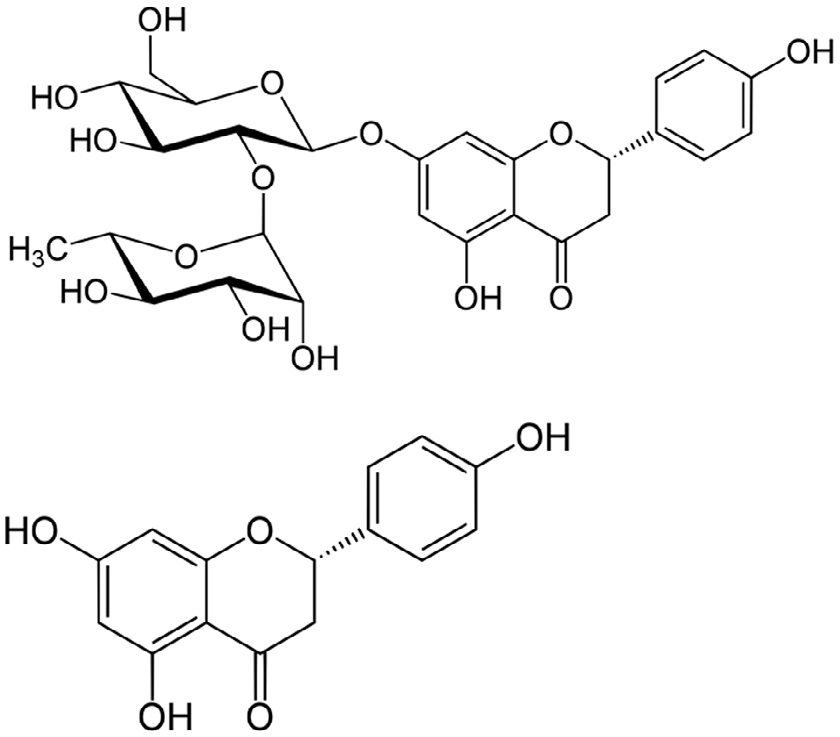
Structures of naringin (*top*) and naringenin (*bottom*).

In 2011 Russo and coworkers in Italy first showed the relevance of effective formulation of otherwise poorly soluble naringin (4′, 5,7‐trihydroxyflavanone‐7‐rhamnoglucoside, **Figure** **5**) for activity in CF treatment based on reduction of hyperinflammatory status of CF cell lines.^[^ ^22^ ^]^

In detail, a naringin‐leucine (N‐Leu) powder (obtained by spray‐drying an aqueous solution of leucine and naringin in H_2_O:EtOH) dissolved in the cell culture medium and immediately administered to both CF and normal bronchial epithelial cells at a concentration of 30 μM reduced the hyperinflammatory status in cystic fibrosis cell lines via inhibiting the expression levels of IKK*α*, IKK*β*, NF‐*κ*B, and phosphorylation of ERK1/2 kinase in CF airway bronchial epithelial cells.

In CF cell lines, the nuclear factor‐*κ*B (NF‐*κ*B) and mitogen‐activated protein kinase/extracellular signal‐regulated kinase (MAPK/ERK) pathways are overactivated. Defective CFTR function indeed induces the expression of pro‐inflammatory mediators also in absence of any infection, with CF cells showing constitutive NF‐*κ*B hyperactivation and ERK upregulation. Tested in vitro on both CF and normal bronchial epithelial cells, the formulations were able to modulate both NF‐*κ*B and MAPK/ERK pathways in absence of stimulation in bronchial epithelia, with higher effects in CF cells than in normal bronchial cells. Remarkable outcomes showing substantial improvement in vitro pharmacological activity of naringin led the team to conclude to suggest potential use of a dry powder inhaler based on co‐spray dried naringin and leucine ‘as MAPK and NF‐*κ*B inhibitors to treat lung intrinsic inflammation and prevent tissue damages in CF patients’.^[^ ^22^ ^]^

In 2017, Su and coworkers in China confirmed that naringenin, the aglycone of naringin, is a powerful CFTR activator able to stimulate Cl^−^ secretion and demonstrated that the mechanism is mediated by CFTR through a signaling pathway by increasing cAMP content.^[^ ^23^ ^]^

In detail, by conducting in vivo (measuring *I*_SC_ in rat airway epithelium) and in vitro experiments (CFTR expression in Calu‐3 cells), the team showed that naringenin, used at 100 μM concentration dissolved in DMSO, induces in Calu‐3 cells a nearly 20 mA cm^−2^ increase in *I*_SC_ and nearly doubled from 8.86 to 16 μA cm^−2^ the basal *I*_SC_ in isolated rat airway tissue when applied to the basolateral side of rat airway tissue. The flavonoid, furthermore, acts directly on the airway epithelial cells to stimulate Cl‐ secretion, rather than on sub‐epithelial interstitial cells of the airway.

The study further proved that the flavonoid triggers cAMP‐dependent chloride secretion via CFTR transportation in airway epithelia and regulates CFTR expression in an LPS‐induced CFTR downregulation model in vivo, ultimately producing an expectorant effect. The researchers concluded that naringenin might be used for treatment for sputum.

## 3 New Therapeutic Potential of Citrus Flavonoids for the Treatment of Cystic Fibrosis

**Table** **2** summarizes achievements in studies devoted to citrus flavonoids for the treatment of cystic fibrosis. Entries for each flavonoid indicate the experimental model (specifying doses or concentrations and timings) and the observed outcomes.

**Table 2:** Achievements in studies devoted to citrus flavonoids for the treatment of cystic fibrosis.

Flavonoid	Experimental model (concentration and timings)	Observed outcomes	Reference
Apigenin	In vitro experiments with Calu‐3 cells, transepithelial measurements in Calu‐3 monolayers; in vivo measurements of nasal potential difference (30 μM).	CFTR activation: maximal current stimulation elicited by apigenin > 400%; maximal current stimulated by apigenin in transepithelial recordings > 200% of forskolin‐stimulated current. In addition to stimulation, apigenin blocked CFTR in forskolin‐stimulated monolayers at high concentration (100 μM).	[10]
Nobiletin	In vitro experiments with cell line 16HBE14o‐ derived from bronchial surface epithelial cells, and cystic fibrosis human airway epithelial cell line CFBE41o‐ (100 μM).	CFTR activation: via a cAMP/PKA‐dependent pathway: in the presence of 10 μM forskolin, which maximally activated the cAMP‐dependent CFTR chloride channel, the addition of nobiletin (100 μM) to the apical or basolateral membrane of 16HBE14o‐ human bronchial epithelial cells increased *I*_SC_ by 12.03 ± 1.18 and 9.69 ± 1.38 μA cm^−^^2^, respectively. Confirming that the nobiletin‐stimulated Cl^−^ secretion is due to the activation of CFTR, the CF cell line (CFBE41o‐), which lacks functional CFTR, no increase in *I*_SC_ is observed after application of apical nobiletin (100 μM).	[17]
Tangeretin	In vitro experiments with Fischer rat thyroid epithelial (FRT) cells (up to 200 μM) and in vivo experiments with mice (Wistar rats) (up to 250 μM).	CFTR activation: tangeretin (20 and 40 μM) increased *I*_SC_ by 70% and 120%, respectively, in FRT cells expressing CFTR; tangeretin induced a dose‐dependent *I*_SC_ in intact rat colonic mucosa, with maximal effect at 250 μM. Tangeretin further increased CFTR chloride channel activity even after forskolin and IBMX saturated CFTR phosphorylation, suggesting CFTR activation by a direct interaction mechanism.	[16]
Quercetin	In vitro experiments with Calu‐3 cells, transepithelial measurements in Calu‐3 monolayers; in vivo measurements of nasal potential difference (30 μM); and in nasal potential difference measurements in humans.	CFTR activation: maximal current stimulation elicited by quercetin > 250% of forskolin‐stimulated current; maximal current stimulated by quercetin in transepithelial recordings ≈200% of forskolin‐stimulated current. In addition to stimulation, quercetin blocked CFTR in forskolin‐stimulated at high concentrations (>100 μM)	[10]
Kaempferol	In vitro experiments with Calu‐3 cells, transepithelial measurements in Calu‐3 monolayers; in vivo measurements of nasal potential difference (30 μM); and in nasal potential difference measurements in humans.	CFTR activation: maximal current stimulation elicited by kaempferol > 320% of forskolin‐stimulated current; maximal current stimulated by kaempferol in transepithelial recordings ≈200%. In addition to stimulation, kaempferol blocked CFTR in forskolin‐stimulated at high concentration (>100 μM)	[10]
Hesperidin	In vitro experiments with cell cultures from murine nasal septal epithelium (MNSE) and human sinonasal epithelium (HSNE), (1,000 μM).	CFTR activation: significant increase in *I*_SC_ of 12.28 ± 1.08 mA cm^−^^2^ in HSNE stimulated with hesperidin dissolved in DMSO. Flavonoid has not effect on cellular cAMP concentration and CFTR R‐domain phosphorylation. Ciliary beat frequency activation: hesperidin (1.0 mM) delivered to the apical membrane and basal media increased CBF by 2.26 ± 0.16 vs. 1.60 ± 0.21 of the control vehicle (phosphate‐buffered saline) likely due to CFTR‐mediated hydration of the airway surface liquid component of the mucociliary apparatus.	[21]
Naringin	In vitro experiments with CuFi1 and NuLi1 cell lines from human bronchial epithelium of a CF (CuFi1, CFTR *Δ*F508/*Δ*F508 mutant genotype) and a non‐CF subject, respectively, (spray‐dried naringin cosprayed with 5% leucine, 30 μM concentration).	Reduction of hyperinflammatory status of CF cell lines: naringin cosprayed with 5% leucine at 30 mM concentration inhibits both mitogen‐activated protein kinase/extracellular signal‐regulated kinase (MAPK/ERK and nuclear factor‐*κ*B (NF‐*κ*B) pathways) cell signaling pathways required for cytokine production, both of which are over activated in bronchial epithelial cells from CF patients.	[22]
Naringenin	In vitro experiments with Calu‐3 epithelial cells and primary rat airway epithelial cells; in vivo experiments with mice (Sprague‐Dawley rats), (100 μM in DMSO)	CFTR activation: treatment of Calu‐3 cells with naringenin 100 mM drives a nearly 20 mA cm^−^^2^ increase in *I*_SC_ and nearly doubles from 8.86 to 16 μA cm^−^^2^ the basal *I*_SC_ in isolated rat airway tissue when applied to the basolateral side of rat airway tissue. Naringenin acts directly on the airway epithelial cells to stimulate Cl‐ secretion, rather than on sub‐epithelial interstitial cells of the airway. Naringenin modulates the activity of CFTR by increasing adenylate cyclase activity.	[23]

Besides acting as CFTR activator, citrus flavonoids hold a vast and mostly unexplored potential for the treatment of CF.

For example, cystic fibrosis lungs are often infected with chronic *Pseudomonas aeruginosa* biofilms.^[^ ^24^ ^]^ Imparted with powerful antimicrobial activity, whose multitarget antibacterial mechanism includes bacterial membrane disruption and reactive oxygen species generation,^[^ ^25^ ^]^ citrus flavonoids may contribute to the treatment of CF in infected patients. Furthermore, numerous citrus flavonoids exert anti‐inflammatory activity.

For example, Tang and coworkers in China recently reported that hesperetin dissolved in DMSO suppresses the expression of TNF‐*α* and IL‐1*β*, two key proinflammatory cytokines, in RAW 264.7 macrophage cells at low concentrations (12.5–50 μM), whereas naringenin has pronounced inhibitory effects on the expression of genes associated with inflammation even at lower concentrations (12.5–25 μM).^[^ ^26^ ^]^ Studying the effects of hesperetin and naringenin dissolved in DMSO on the expression of inflammation‐related genes in RAW 264.7 cells, the team found that both flavonoids exhibit significant inhibitory effects on the expression of inflammatory genes, in particular of TNF‐*α*, IL‐1*β*, and iNOS inflammatory mediators. TNF‐*α* and IL‐1*β*, two key proinflammatory cytokines, play a pivotal role in the inflammatory response. iNOS also plays a significant role in the inflammatory process through its product, nitric oxide.

As noted by Russo and coworkers in 2011 reporting the anti‐inflammatory activity *in vitro* of naringenin aptly formulated with leucine,^[^ ^22^ ^]^ the lungs of CF patients experience chronic hyperinflammation even when not affected by bacterial infection so that reduction of inflammation may prevent tissue damages in CF patients. A recent review of numerous studies devoted to the use of citrus flavonoids in the treatment of atherosclerosis,^[27^^,^ ^10^ ^]^ a chronic inflammatory disorder accompanied with oxidative stress, concluded that markers of chronic inflammation such as interleukins, tumor necrosis factor‐*α*, nuclear factor‐*κ*B, and nitric oxide signaling, as well as oxidative stress markers like superoxide dismutase and glutathione, were all normalized upon administration of citrus flavonoids.^[^ ^27^ ^]^

In general, citrus flavonoids have extremely low solubility in water, leading to poor oral bioavailability.^[^ ^28^ ^]^ The thesis of this work is that newly developed formulations of citrus flavonoids hold significant potential for developing a multitarget treatment of cystic fibrosis symptoms combining in a single treatment antimicrobial, anti‐inflammatory, CFTR‐stimulating, and immunomodulatory therapy, as may be required by a genetic pathology eventually causing lung inflammation and bacterial infection reinforcing each other in a vicious cycle.

Said otherwise, likewise to what has been done for most nutraceuticals based on citrus flavonoids,^[^ ^29^ ^]^ the development of citrus flavonoids into effective drugs for the treatment of CF requires the development of delivery pharmaceutical technologies by which to enhance the limited bioavailability of these compounds due to their poor solubility in water.^[^ ^30^ ^]^

## 4 Conclusions

In summary, in the first study in 1998, Illek and Fisher showed that selected citrus flavonoids such as apigeninm quercetin and kaempferol dissolved in DMSO are able to activate CFTR‐mediated Cl^−^ currents both in vivo and in vitro using single Calu‐3 cells and in vivo measurements of nasal potential difference. More than a decade later, new work from researchers based at the University of Alabama confirmed that quercetin activates CFTR‐mediated anion transport in respiratory epithelia in vitro and in vivo and found that quercetin does not produce detectable phosphorylation of the isolated CFTR R‐domain. The team of Rowe and coworkers also suggested an activation mechanism independent of channel phosphorylation, producing a dose‐dependent hyperpolarization of the nasal potential difference also in normal human subjects. Nine months later, Woodwarth and coworkers from the same institution reported that hesperidin dissolved in DMSO is a CFTR‐dependent Cl^−^ secretagogue in murine and human nasal airway cells in vitro and that the compound is an activator of ciliary beat frequency.

In 2010, Rowe's team reported analogous (though lower) ciliary beat frequency stimulation activity for quercetin dissolved in DMSO. The researchers concluded that this finding had therapeutic implications for chronic rhinosinusitis recommending clinical studies ‘targeting topical administration to the sinuses, including individuals with relative deficiency in CFTR activity’.^[^ ^19^ ^]^

Showing how leucine and naringin first dissolved in a water/ethanol 7:3 mixture and then co‐spray dried substantially improved in vitro pharmacological activity of naringin based on reduction of hyperinflammatory status, Russo and coworkers in Italy first showed in 2011 the relevance of effective formulation of otherwise poorly soluble naringin for activity in CF treatment.

Between 2013 and 2014, scholars in China led by Yang reported that both nobiletin and tangeretin, two flavonoids abundant in several citrus fruits peel, are CFTR chloride channel activators whose activation mechanism involves direct interacton with the protein. In both cases, the team concluded that two flavonoids might have potential use for the treatment of CFTR‐related diseases like CF and bronchiectasis. In 2015, Ko and coworkers in Hong Kong confirmed that nobiletin stimulates transepithelial Cl^−^ secretion across human bronchial epithelia, and that the mechanism involves activation of adenylate cyclase‐ and cAMP/PKA‐dependent pathways, leading to activation of apical CFTR Cl^−^ channels.

Finally, in 2017, Su and coworkers in China confirmed that naringenin, a dihydroflavone abundant in the peel of grapefruit, has the ability to stimulate chloride secretion and demonstrated that the mechanism is mediated by CFTR through a signaling pathway by increasing cAMP content, concluding that naringenin may act as treatment for sputum.

In conclusion, the analysis of the first research achievements concerning the use of citrus flavonoids for the treatment of CF symptoms suggests relevant early findings. Flavones, flavanols, and flavanones show activity as CFTR activators and, in certain cases also as ciliary beat frequency activators. The CFTR activation mechanism does not involve phosporylation of the CFTR R‐domain, but rather direct binding to a stimulatory site eventually increasing adenylate cyclase activity.

Limitations of the current research on citrus flavonoids for treating CF include the variability of research design, the use of different cell lines, and the lack of long‐term toxicity studies. All said limitations, however, will be taken into account designing and carrying out preclinical research. Further supporting the need to reinvigorate research on citrus flavonoids to treat CF, indeed, recent molecular docking and molecular dynamics calculations clearly showed that quercetin binds to the mutated CFTR protein structure active sites (via hydrophobic bonds, hydrogen bonds, and electrostatic interactions), remaining bound to the target protein favorably and dynamically, thereby inhibiting the effect of mutated CFTR protein through improved trafficking and restoration of original function.^[^ ^31^ ^]^

Following preclinical research, part of which summarized in this account, clinical studies will be required to assess the efficacy, safety, and optimal usage of new therapeutic interventions based on novel formulations based on citrus flavonoids in the treatment of CF. This, in its turn, will require phase I (first‐in‐human studies to evaluate safety, dosing, and pharmacokinetics), phase II (randomized controlled trials, RCTs, in a larger population), and phase III (multicenter RCTs to confirm efficacy) clinical trials.

It is relevant, in this context, that citrus flavonoids are widely approved ingredients in numerous food, nutraceutical, and pharmaceutical products. From diosmin combined with hesperidin used as active pharmaceutical ingredient (API) in drug used to treat chronic venous insufficiency^[^ ^32^ ^]^ to mixtures of citrus flavonoids used in antibacterial formulations,^[^ ^33^ ^]^ numerous pharmaceutical products use citrus flavonoids as APIs.

Reinforcing the thesis of this study is the fact that even in the case of diosmin combined with hesperidin for the treatment of chronic venous insufficiency, micronization of diosmin was necessary to enhance the pharmacodynamic and clinical activities in comparison to an equivalent dose of nonmicronized diosmin.^[^ ^34^ ^]^ Furthermore, it is encouraging that RCTs have already proven the beneficial effect of citrus flavonoid supplementation in enhancing for example the endothelial function.^[^ ^35^ ^]^

In general, the interest for the therapeutic properties of citrus flavonoids has dramatically expanded, and numerous clinical studies focusing on these flavonoids for the treatment of serious pathologies, including metabolic diseases, have been lately planned. As noted by Nawaz and coworkers, ‘these trials will likely explore different flavonoids, combinations thereof, and their interactions with other treatments…the coming years promise a surge of valuable insights into the therapeutic potential of flavonoids’.^[^ ^36^ ^]^

This study, in conclusion, will hopefully contribute to promote the first pre‐clinical and clinical studies focusing on flavonoids sourced by the peel of Citrus fruits for the treatment of the most common inherited disease among Caucasians.^[^ ^37^ ^**,**^ ^38^ ^]^

## Conflict of Interest

The authors declare no conflict of interest.
